# Characterizing patterns of genomic variation in the threatened Utah prairie dog: Implications for conservation and management

**DOI:** 10.1111/eva.13179

**Published:** 2020-12-21

**Authors:** Rachael M. Giglio, Tonie E. Rocke, Jorge E. Osorio, Emily K. Latch

**Affiliations:** ^1^ Department of Biological Sciences University of Wisconsin‐Milwaukee Milwaukee WI USA; ^2^ USGS National Wildlife Health Center Madison WI USA; ^3^ Department of Pathobiological Sciences School of Veterinary Medicine University of Wisconsin‐Madison Madison WI USA

**Keywords:** conservation genomics, genetic drift, genotype–environment associations, local adaptation, population divergence, Utah prairie dog

## Abstract

Utah prairie dogs (*Cynomys parvidens*) are federally threatened due to eradication campaigns, habitat destruction, and outbreaks of plague. Today, Utah prairie dogs exist in small, isolated populations, making them less demographically stable and more susceptible to erosion of genetic variation by genetic drift. We characterized patterns of genetic structure at neutral and putatively adaptive loci in order to evaluate the relative effects of genetic drift and local adaptation on population divergence. We sampled individuals across the Utah prairie dog species range and generated 2955 single nucleotide polymorphisms using double digest restriction site‐associated DNA sequencing. Genetic diversity was lower in low‐elevation sites compared to high‐elevation sites. Population divergence was high among sites and followed an isolation‐by‐distance model. Our results indicate that genetic drift plays a substantial role in the population divergence of the Utah prairie dog, and colonies would likely benefit from translocation of individuals between recovery units, which are characterized by distinct elevations, despite the detection of environmental associations with outlier loci. By understanding the processes that shape genetic structure, better informed decisions can be made with respect to the management of threatened species to ensure that adaptation is not stymied.

## 1 INTRODUCTION

As a result of habitat loss and fragmentation, threatened species often occur in small, isolated populations. Genetic variation can be rapidly eroded in these small populations by genetic drift, a process that goes unmitigated in isolated populations without gene flow (Frankham, 1996). A lack of genetic variation weakens species viability, stifling the evolutionary potential of a species and constraining adaptation to local environmental conditions (Barrett & Schluter, 2008; Savolainen et al., 2013). As global environmental change intensifies (Urban, 2015; Wiens, 2016), the ability of a species to adapt to changing local conditions will be increasingly central to its long‐term viability.

Conservation and management activities that facilitate the retention of genetic variation, evolutionary potential, and adaptability will further help species avoid extinction. For example, translocations, used to bolster declining populations, can be improved by incorporating genetic data to tailor translocation actions to outcomes that boost genetic variation. Selecting source populations that are genetically appropriate for the target population (Johnson et al., 2010), prioritizing target populations with low genetic variation (Whiteley et al., 2015), or gauging incorporation of source genotypes (Bateson et al., 2014; Latch & Rhodes, 2005; Mulder et al., 2017) can improve efforts to retain genetic variation. Conservation and management actions could further improve the evolutionary potential and adaptive capacity of populations by incorporating data from studies of adaptive variation, especially if those actions include translocations, genetic rescue, or assisted gene flow (Flanagan et al., 2018; Funk et al., 2018). Advances in genomics for nonmodel species means that researchers can generate broad coverage and high‐resolution genomic data for an increasing number of species. Genomic data can be used to survey both adaptive and neutral genetic variation and can be incorporated into conservation policy to improve long‐term species viability against changing environments and exposure to new diseases (Flanagan et al., 2018; Funk et al., 2018).

In this study, we use a population genomics approach to understand the maintenance of genetic variation in the threatened Utah prairie dog (*Cynomys parvidens*). The Utah prairie dog is one of five species of prairie dog found in North America and is listed as Threatened under the United States' Endangered Species Act and as Endangered by the IUCN Red List of Threatened Species (Roach, 2018). Due to heavy range‐wide eradication campaigns during the 20th century, as well as ongoing habitat loss/fragmentation and epizootic outbreaks of plague, the Utah prairie dog has been reduced from a range‐wide estimate of 95,000 individuals (estimated in the 1920s) to approximately 8969 today (total 2018 spring count of adults) (Collier & Spillett, 1973; Kavalunas & Day, 2018). In 1972, a recovery plan for the Utah prairie dog was enacted that focused on translocating individuals from private land to protected public lands (McDonald, 1993; United States Fish & Wildlife Service, 2012). Today, Utah prairie dogs exist in small, isolated populations, making them less demographically stable and more susceptible to the erosion of genetic variation through genetic drift (Gilpin & Soulé, 1986; Wright, 1931). Further, Utah prairie dogs are highly social mammals that live in small family groups called coteries (consisting of one or two unrelated adult males, a group of related females, and their young; Hoogland, 2006), with adjacent coteries forming a colony. In contrast to many mammalian species that exhibit natal dispersal, Utah prairie dogs rarely leave their natal coteries unless nearly all of the individuals in a coterie are gone (Hoogland, 2013), for example following an outbreak of plague. Any such dispersal that does occur is likely male‐biased and to nearby coteries, often within the same colony (Hoogland, 2013). Due to prairie dog behavior coupled with the fragmented structure of their habitat patches, these colonies likely exhibit metapopulation dynamics (Brown et al., 2016).

Translocations are a common practice in the management of prairie dogs (United States Fish & Wildlife Service, 2012). This is an effective tool to combat the loss of genetic variation experienced through a history of population bottlenecks, eradication campaigns, disease, and limited natural gene flow. However, a large degree of variation in habitat exists across the Utah prairie dog species range. Utah prairie dogs can be found from valley floors to montane habitats, with elevations ranging from roughly 1.5 to 2.9 km above sea level (Roach, 2018). Combined with a lack of natural gene flow, this habitat heterogeneity could encourage local adaptation (Blanquart et al., 2013). Under this scenario, translocating individuals from different areas of the species range could introduce maladaptive genes to other colonies, leading to outbreeding depression. Even though fears of outbreeding depression may be inflated (Frankham et al., 2011; Ralls et al., 2018), translocations between locally adapted populations could still have potentially disastrous consequences for the viability of prairie dogs. By characterizing not only neutral genetic markers, but also those under selection, we may avoid stifling the evolutionary potential of Utah prairie dogs.

The objective of this study was to use a population genomics approach to investigate the effect of gene flow, genetic drift, and divergent selection on the maintenance of genetic variation in Utah prairie dogs across their species range. To accomplish this objective, we carried out five aims. First, we characterized patterns of genetic structure and gene flow among Utah prairie dog populations. Second, we evaluated the impact of sex‐biased dispersal on the maintenance of genetic variation by identifying differences in patterns of genetic structure and gene flow for females and males separately. Third, we characterized the impact of genetic drift on the erosion of genetic variation and genetic differentiation. Fourth, we identified loci under divergent selection and compared patterns of population divergence at these loci against neutral loci. Fifth, we examined how the environment might influence local adaptation by identifying genotype–environment associations (GEAs). Our genome‐wide approach allows us to harness information in both neutral and adaptive loci to tailor conservation activities to maximize the success of recovery efforts without incurring the potentially substantial costs that could result from translocating locally adapted individuals.

## 2 METHODS

### 2.1 Generating the SNP dataset

**FIGURE 1:**
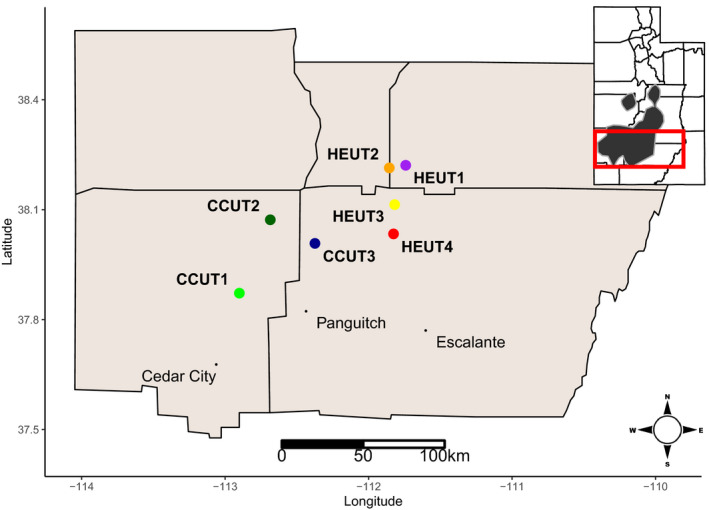
Map of Utah prairie dog sampling locations across the species range (shown in dark gray on the Utah state inset map). Prairie dogs were sampled from two locations at each site (three sites near Cedar City, Utah [CCUT] and 4 high‐elevation sites on the Awapa Plateau [HEUT])

**FIGURE 2:**
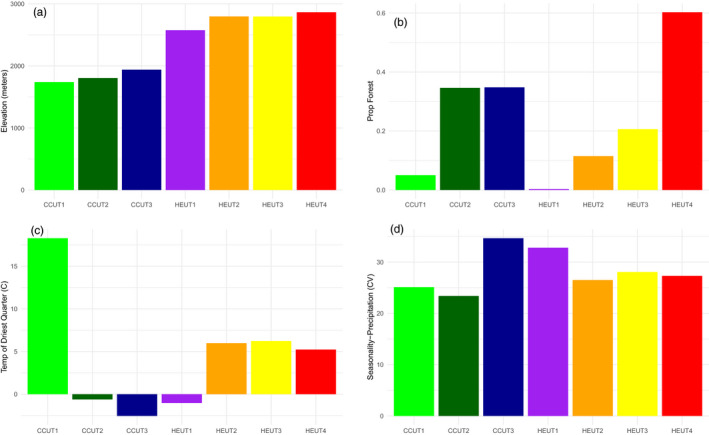
Landscape (Elevation [NAVD88], in meters) and the proportion of forest within a 5 km buffer around sampling sites [PropForest]) and climatic variables (temperature of the driest quarter °C [Bio9] and the precipitation seasonality [coefficient of variation; Bio5] varied across sampling sites. These variables were found to be uncorrelated and were included in the redundancy analysis to identify genotype–environment associations

We trapped Utah prairie dogs and collected hair and whiskers during a field trial of a sylvatic plague vaccine (Rocke et al., 2017). Samples were collected in 2014 from two plots within each site (assumed to be a single prairie dog colony), with plots located in proximity (0.15–2.10 km). Due to a high degree of movement between plots observed during the vaccine field trial, plots were treated as a single site for our analyses. Individuals were sampled throughout the Utah prairie dog range at three sites near Cedar City and Panguitch, Utah (CCUT) and four high‐elevation sites within the Awapa Plateau (HEUT; Figure 1). From the total estimated area of occupancy for Utah prairie dogs (2800 ha; Roach, 2018), we sampled 29.2 ha for CCUT sites and 54.7 ha for HEUT sites (Rocke et al., 2017). These sites are within the three recovery units (RUs) of Utah prairie dog (CCUT1 and CCUT2 sites are within the West Desert RU, the CCUT3 is within the Pansaugunt RU, and all of the HEUT sites are within the Awapa RU) and varied in elevation with CCUT and HEUT sites at a mean of 1.83 and 2.76 km (NAVD88), respectively (CCUT1 = 1.74 km; CCUT2 = 1.80 km; CCUT3 = 1.94 km; HEUT1 = 2.58 km; HEUT2 = 2.80 km; HEUT3 = 2.80 km; and HEUT4 = 2.86 km; Figure 2), approaching the full extent of elevational distribution for this species. These RUs are separated by biogeographic barriers—Cedar Mountain, the Hurricane Cliffs, and Markagunt Plateau are between West Desert RU and Pansaugunt RU, and the Escalante Mountains, the East Fork Sevier River Gorge, and Parker Mountain are between the Pansaugunt RU and the Awapa Plateau RU. Variation in precipitation (mean annual precipitation CCUT = 278.0 mm; HEUT = 387.5 mm for years 1965–1978), temperature regimes (mean annual temperature CCUT = 9.0°C; HEUT = 3.5°C), and habitat exists among sites (Figure 2; Figures S1 and S2; Hijmans et al., 2005; Homer et al., 2012). DNA was extracted from the hair and whiskers using the Zymo universal spin column‐based tissue extraction kit (Zymo Scientific) following the manufacturer's feather and hair follicle protocol. We sequenced 15–59 individuals (mean = 33.86 individuals, total = 237) per site (Table 1).

**TABLE 1:** Measures of genetic variability in Utah prairie dogs (*Cynomys parvidens*) for (a) each of the sampled sites and (b) the two putative populations identified using both STRUCTURE and DAPC based on 2955 variable SNP loci

	*N*	*N*_M_	*N*_f_	*H*_O_	*H*_E_	A_R_	*A*_P_	*F*_IS_ (95% CI)	*N*_e_ (95% CI)
(a) Sampled sites									
CCUT1	40	28	12	0.11	0.09	1.26	1	−0.21 (−0.24 to −0.19)	25.4 (16.7–44.4)
CCUT2	58	29	29	0.11	0.09	1.29	2	−0.21 (−0.23 to −0.20)	8.6 (2.7–44.7)
CCUT3	21	14	7	0.15	0.13	1.39	8	−0.18 (−0.21 to −0.15)	Infinity
HEUT1	44	20	21	0.31	0.29	1.86	17	−0.07 (−0.10 to −0.04)	12.7 (6.2–30.3)
HEUT2	24	15	9	0.23	0.20	1.57	0	−0.14 (−0.20 to −0.09)	10.6 (6.9–16.9)
HEUT3	13	5	8	0.29	0.25	1.74	0	−0.15 (−0.21 to −0.10)	6.3 (3.0–11.4)
HEUT4	33	13	20	0.23	0.26	1.74	0	0.11 (0.05–0.16)	2.2 (1.3–7.3)
(b) Putative populations									
CC	119	71	48	0.12	0.12	1.54	111	0.04 (−0.01 to 0.08)	
HE	114	53	58	0.27	0.30	1.96	1326	0.10 (0.07–0.12)	

Abbreviations: *A*_P_, number of private alleles; *A*_R_, allelic richness; *F*_IS_, inbreeding with 95% confidence intervals (95% CI); *H*_E_, expected heterozygosity; *H*_O_, observed heterozygosity; *N*, sample size; *N*_e_, estimated effective population size with 95% confidence intervals (95% CI); *N*_f_, number of females sampled; *N*_M_, number of males sampled; SNP; single nucleotide polymorphism.

To generate single nucleotide polymorphisms (SNPs), samples with >300 ng of total genomic DNA, quantified using a Qubit 2.0 Fluorometer (Invitrogen), were used for double digest restriction site‐associated DNA sequencing (ddRAD; Peterson et al., 2012). Genomic DNA was digested using the restriction enzymes *HindIII* and *NlaIII*, barcoded, and size selected for 250–500‐bp fragments using a Pippin Prep (Sage Sciences). Fragments were paired‐end sequenced on an Illumina NovaSeq6000 at Texas A&M AgriLife Genomics. We aligned sequences to a Gunnison's prairie dog (*Cynomys gunnisoni*) genome (Tsuchiya et al., 2020) using the BWA short‐read aligner with default parameters and the MEM alignment algorithm (Li & Durbin, 2009). Contigs were assembled using the program STACKS v.1.48 software (Catchen et al., 2011, 2013), following the proposed workflow outlined by Rochette and Catchen (2017).

After calling SNPs, several additional quality control measures were taken. First, in cases where more than 1 SNP per contig was present, only the first (most 5′) SNP was used. Second, only loci represented in 80% or more of individuals were retained. Third, only loci present in all 14 sampling locations were retained. Fourth, individuals missing >30% of data (*n* = 4) were removed (calculated using VCFtools v.0.1.16; Danecek et al., 2011). Fifth, because low‐frequency alleles may represent PCR errors, we removed loci with minor allele frequencies <0.05. Sixth, we removed potential paralogs by excluding loci with an observed heterozygosity exceeding 0.7 using the populations module of STACKS, and loci with a depth of coverage greater than twice the mode of the depth of coverage for each locus using R (O'Leary et al., 2018; Willis et al., 2017). Paralogous loci can skew common downstream analyses for population genomics by artificially inflating levels of heterozygosity (Willis et al., 2017).

### 2.2 Characterizing the patterns of genetic structure, gene flow, and sex‐biased dispersal

To visualize genetic divergence, we used a principal component analysis (PCA). We used two methods to determine the number of genetic clusters (*K*) present in our sampling sites: the Bayesian clustering program STRUCTURE v2.3.4 (Pritchard et al., 2000) and a multivariate approach using discriminant analysis of principal components (DAPC) in the R package adegenet (Jombart & Ahmed, 2011; Jombart et al., 2010). We ran STRUCTURE with a Markov chain Monte Carlo (MCMC) burn‐in of 100,000 steps followed by 100,000 steps for inference clustering using the admixture model with correlated allele frequencies. For each value of *K*, we completed 10 replicates. For STRUCTURE, we used the alternative prior for population‐specific ancestry (*α* = 1) because we had unequal sampling among sites (Wang, 2017). In order to accurately resolve the number of genetic clusters (*K*) using STRUCTURE, we used a combination of the LnP(*D*) and Δ *K* as outlined in Janes et al. (2017) calculated using STRUCTURE HARVESTER v.0.6.93 (Earl & vonHoldt, 2012). We also adopted a ‘hierarchical STRUCTURE analysis’ approach where each genetic cluster was analyzed iteratively in a new STRUCTURE run in order to gauge substructure (Vähä et al., 2007). We used the program CLUMPP 1.1.2 (Jakobsson & Rosenberg, 2007) to assign individuals to genetic clusters using *q*‐values from STRUCTURE. For the DAPC, the analysis was first performed unsupervised (no prior knowledge of groups) using the sequential *K*‐means clustering algorithm executed through the find.clusters function in adegenet (Jombart & Ahmed, 2011; Jombart et al., 2010). We then performed the DAPC analysis supervised by using the Bayesian information criterion (BIC) to determine the final value of *K*.

To determine patterns of gene flow, we calculated the pairwise genetic differentiation between sites using Weir and Cockerham's *F*_ST_ and Jost's *D* using the R packages hierfstat (Goudet & Jombart, 2015) and mmod (Winter, 2012), respectively. We tested for significant population divergence using both *F*_ST_ and Jost's *D* with 1000 random permutations and corrected *p*‐values for multiple comparisons using a false discovery rate (FDR) correction (used the ‘p.adjust’ function in R v3.5.2; Benjamini & Hochberg, 1995; R Core Team, 2018). To test for sex‐biased dispersal, we repeated the above analyses on males and females separately. We estimated the effective number of migrants (*N*_M_) based on the number of private alleles using the R package genepop (Barton and Slatkin, 1986; Rousset, 2008). We estimated relative migration rates among sampling sites using *N*_M_ with the divMigrate function in the R package diveRsity and identified significant migration rates using 10,000 bootstrap iterations (Alcala et al., 2014; Keenan et al., 2013; Sundqvist et al., 2016). Migration networks were created using the R package qgraph (Epskamp et al., 2012). Values <1 indicate that populations will diverge due to drift.

### 2.3 Characterizing the effect of genetic drift

Genetic drift erodes standing genetic variation where rare alleles face a greater chance of being lost due to random chance. As populations decrease in size and become isolated, the effects of genetic drift grow in significance, which accelerates the erosion of genetic variation. Thus, populations in which genetic drift is the primary driver of genetic structure are predicted to have (a) less genetic variation, (b) fewer private alleles (or number of unique alleles for a specific location), and (c) a higher degree of inbreeding than populations in which genetic structure is shaped by other mechanisms. We characterized genetic variation in each site as well as each genetic cluster by measuring expected heterozygosity (*H*_E_), observed heterozygosity (*H*_O_), allelic richness (*A*_R_), the number of private alleles (A_P_), and inbreeding (F_IS_). We calculated *H*_E_, *H*_O_, and *A*_P_ using the R package poppr v.2.8.2 (Kamvar et al., 2014) and *A*_R_ and *F*_IS_ using the R package diveRsity, after which 95% confidence intervals were calculated using 1000 bootstrap iterations (Keenan et al., 2013).

We estimated the effective population size (*N*_e_) and tested for evidence of recent genetic bottlenecks for each site, as these factors significantly impact the rate at which genetic variation is lost as well as the rate of increase of inbreeding and genetic drift (Banks et al., 2013; Charlesworth, 2009; Gasca‐Pineda et al., 2013). We estimated *N*_e_ for each site using the linkage disequilibrium (LD) method implemented in the program NeEstimator v2.1 (Do et al., 2014). One assumption to calculate *N*_e_ using the LD method is that linkage disequilibrium at independent loci in randomly mating, closed populations comes exclusively from genetic drift (Hill, 1981). To meet this assumption, we created a neutral set of loci by excluding loci potentially under the influence of natural selection; those identified as outliers by either BayeScan or PCAdapt for this analysis (see next section for details).

We tested each of the seven prairie dog sampling sites as well as each genetic cluster for evidence of genetic bottlenecks using the program Bottleneck v.1.2.02 (Cornuet & Luikart, 1996; Piry et al., 1999). Like the estimation of *N*_e_, only neutral loci were used to test for evidence of bottlenecks. We used the infinite alleles model (IAM) and tested for significant heterozygosity excess compared to the level predicted under mutation‐drift equilibrium using standardized differences tests (Cornuet & Luikart, 1996).

### 2.4 Neutral loci versus loci under selection

Outlier loci were detected via a Bayesian method conducted in BayeScan 2.1 (Foll & Gaggiotti, 2008) and a nonconstrained ordination method executed in the R package PCAdapt (Luu et al., 2019). BayeScan is based on the multinomial‐Dirichlet model and identifies differences in allele frequencies between subpopulations (we used sampling sites) and the common gene pool of all subpopulations (measured as a subpopulation specific *F*_ST_ coefficient). For the BayeScan method of outlier detection, we defined populations as the sampling sites (*n* = 7) and used the default parameter settings (prior odds for neutrality = 10; odds are that 1 locus is under selection for every 10 neutral loci). We directly controlled the false discovery rate (FDR) by setting the target FDR (*q*‐value) to limit the proportion of false positives to 5% (Foll & Gaggiotti, 2008). For the PCAdapt method, predefined populations are not required. We specified the *K* parameter (the number of PCs to retain) in PCAdapt as 5 based on where the amount of variation explained by each PC decreases (identified as the point of inflection in the PCAdapt ‘scree plot’). We defined outlier loci as those with *q*‐values <0.05, meaning that 5% or less of loci identified as outliers are potentially false positives. To generate the set of outlier loci, we retained loci that were identified as under divergent selection in both BayeScan and PCAdapt methods. We compared this outlier dataset to the neutral set of loci to assess the relative contribution of genetic drift and selection in shaping patterns of genetic structure. Specifically, we compared patterns of genetic structure for outlier and neutral loci using the program STRUCTURE and by using DAPC in adegenet (Jombart & Ahmed, 2011; Jombart et al., 2010). Gene flow was also estimated for each set of loci using pairwise Jost's *D* and *F*_ST_. To determine if geographic distance alone drives patterns of genetic structure, we tested for isolation by distance (IBD) in the full genetic dataset (neutral and outlier loci), neutral loci only, and outlier loci only using Mantel tests between genetic and geographic distance carried out in the R package adegenet (Jombart & Ahmed, 2011; Jombart et al., 2010). We calculated genetic distance using linearized pairwise *F*_ST_ values (*F*_ST_/(1 − *F*_ST_)) and geographic distance was calculated as the pairwise Euclidean distance between sites (km) using the pointDistance function in the R package “raster” (Hijmans, 2019). Significance of Mantel tests was assessed based on 999 replicates.

### 2.5 Selection and environmental associations

We conducted a GEA analysis using a multivariate ordination method, redundancy analysis (RDA), implemented in the R package ‘vegan’ (Forester et al., 2018; Oksanen et al., 2019). This was conducted independent of previous outlier tests and used the full panel of loci to (a) identify outlier loci and (b) identify associations between environmental variables and outlier loci. For the environmental comparisons, we used land cover data from the National Landcover Database (NLCD; resolution = 30 m × 30 m; Homer et al., 2012), the National Elevation Dataset (resolution = 1 arc‐second; Gesch et al., 2018), and climatic variables from Worldclim (resolution = 30 arc‐seconds; Hijmans et al., 2005). For the NLCD data, we first reduced the number of categories to 7 (Open Water, Developed = Developed [low, medium, and high]; Forest = Evergreen, Deciduous, and Mixed Forests; Wetland = Woody Wetlands and Emergent Herbaceous Wetlands; Shrub = Shrub/Scrub; Barren Land; and Crop = Cultivated Crops, Hay/Pasture, and Herbaceous). Because prairie dog sampling coordinates represent the center of a larger sampling area, we characterized each site by the proportion of each landcover type within a 5‐km buffer around the coordinate point (Figure S1). Only the dominant landcover types were retained (represented >5% of landcover at any site; Forest, Shrub, Crop). To avoid multicollinearity, we used Pearson's correlations to remove variables that were correlated with an |*r*| of 0.7 or higher prior to performing the RDA. Predictor variables in the RDA model were further pruned based on their variance inflation factors (VIFs; multicollinearity was assumed if VIF >4). Using the RDA, we identified outlier loci and the environmental variables most associated with those loci (Capblancq et al., 2018; Forester et al., 2018). To prepare the genetic data for the RDA, we replaced missing data with the most common genotype across individuals and converted the full SNP dataset to allele counts. We used an analysis of variance (ANOVA) to quantify how well the full RDA model as well as each RDA axes explained genetic variation using the ANOVA.cca function in the R package vegan with 999 permutations (Legendre et al., 2011; Oksanen et al., 2019). Outlier loci were identified as those with loadings ± 3 standard deviations away from the mean loading. We used all of the significant constrained axes of the partial RDA for outlier detection (SNPs that load heavily on the axes are more likely to be under selection, *p* < 0.05). To infer how each SNP relates to the environmental variables, we identified the environmental variable that was most strongly correlated to each outlier SNP based on correlation coefficients. To illustrate these environment‐SNP associations, we created triplots of all RDA axes using the R package vegan (Oksanen et al., 2019). For the triplots, symmetrical scaling (using the square root of eigenvalues) was used for both SNP and individual scores.

## 3 RESULTS

### 3.1 Generating the SNP dataset

After initial filtering steps, a total of 3549 variable SNP loci were retained. An additional 594 loci were removed due to exceptionally high depth of coverage, indicating potential paralogs (2× the mode of the mean depth of coverage for each locus; mode = 17.55). The final genomic dataset contained 2955 variable SNP loci with a mean depth of coverage of 20.08 for all individuals (mean per locus depth of coverage ranged from 7.68 to 35.58; Figure S3). Four individuals with a high amount of missing data (>30%) were removed from the dataset, leaving a total of 233 individuals used for subsequent analyses (13–58 individuals per site, mean = 33.29). These 233 individuals used for analyses had a mean of 4.2% missing data (Figure S4).

### 3.2 Characterizing patterns of genetic structure, gene flow, and sex‐biased dispersal

**FIGURE 3:**
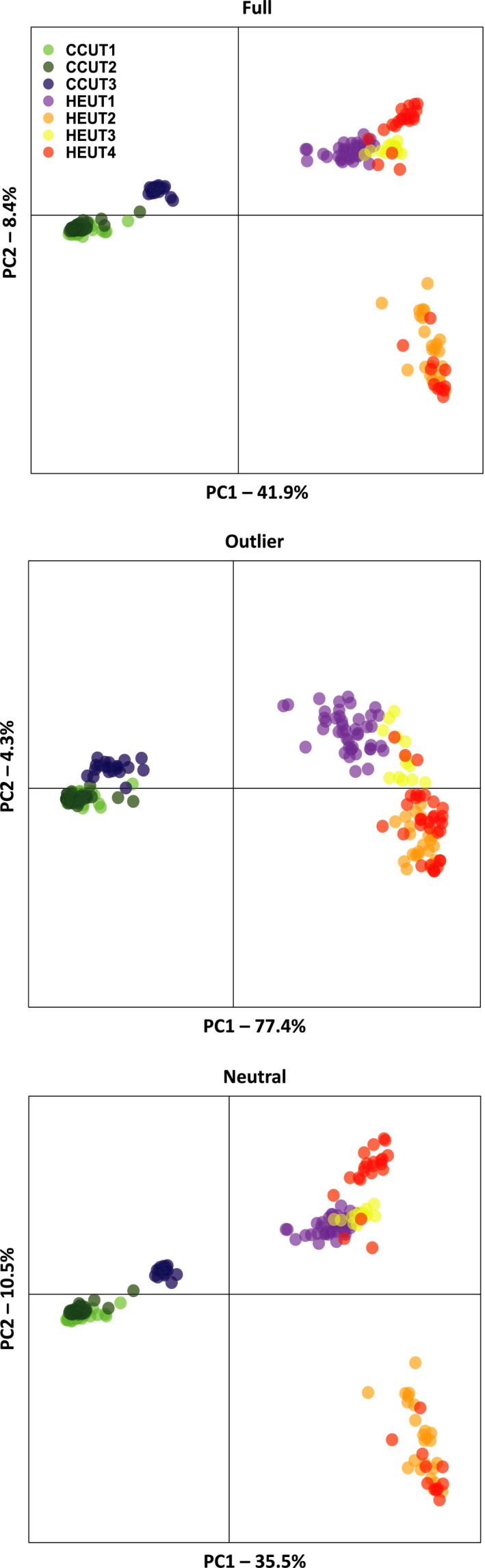
Principal component analysis (PCA) to characterize genetic differentiation among Utah prairie dogs using all single nucleotide polymorphism (SNP) loci (Full), only SNP loci identified as outliers in both BayeScan and PCAdapt (Outlier), and only neutral SNP loci (Neutral). Colors correspond to sites (CCUT1, CCUT2, CCUT3, HEUT1, HEUT2, HEUT3, and HEUT4)

Differentiation among sampling sites was observed using PCA (Figure 3). The most supported number of genetic clusters (*K*) was two, using both the program STRUCTURE (based on Ln(*K*) and the ∆*K* method) and the unsupervised clustering method using DAPC (Figures S5 and S6). In the two clusters, all individuals from the CCUT sites grouped to make one genetic cluster and all individuals from HEUT sites grouped to make the second genetic cluster. When using a supervised clustering approach for DAPC, additional clustering solutions (*K* = 2–4) were informative for describing genetic structure based on BIC (Figure S7). For example, under a *K* of four for DAPC, CCUT3 formed a separate genetic cluster and an additional genetic cluster included all individuals from HEUT2 and 11 individuals from HEUT4 (Figure S8). This pattern was also observed using the hierarchical clustering approach in STRUCTURE (Figure S6).

**FIGURE 4:**
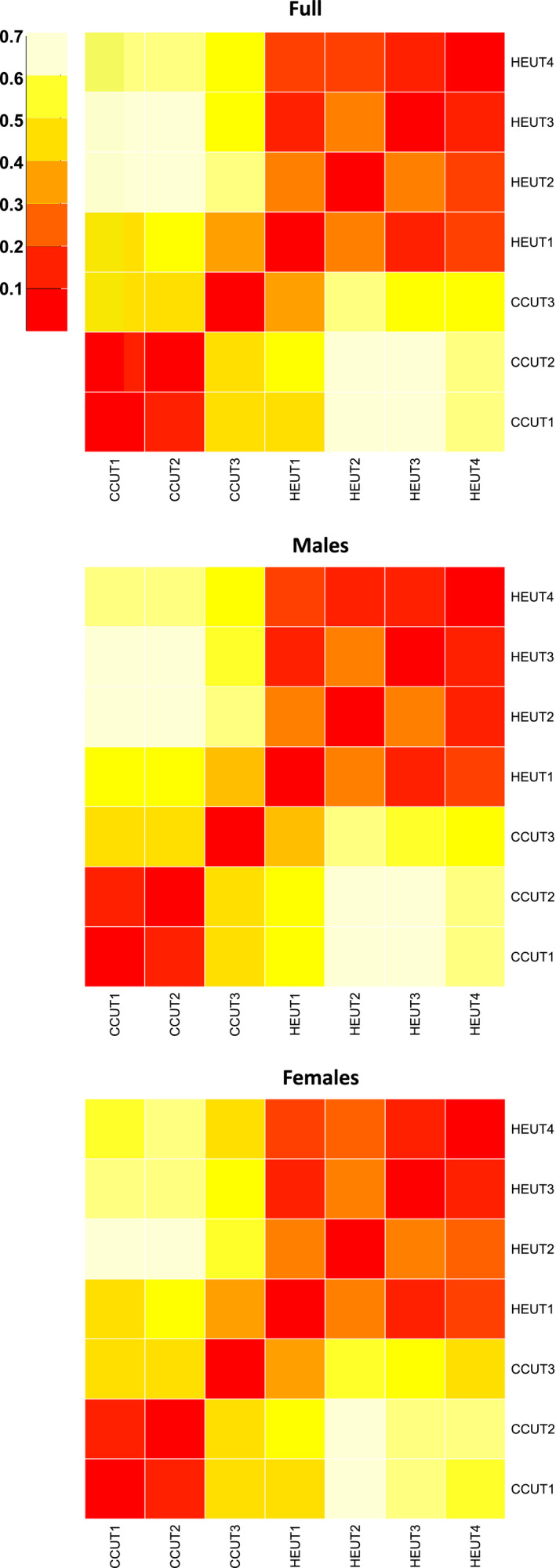
Population differentiation based on pairwise population divergence (*F*_ST_) across Utah prairie dog sites for all individuals (Full); only males (Males); and only females (Females). Warmer colors indicate lower *F*_ST_ (less differentiation) while cooler colors indicate higher *F*_ST_ (more differentiation)

We found similar patterns of population differentiation with pairwise values of *F*_ST_ and Jost's *D* (Table S2), so only values of *F*_ST_ are reported. We detected significant genetic differentiation among sampling locations based on *F*_ST_ (Figure 4, Table S2). Particularly, a high degree of differentiation was observed between the CCUT sites and the HEUT sites. Among those CCUT to HEUT comparisons, the lowest *F*_ST_ values were between the CCUT3 and the HEUT1 sites. Further, the CCUT3 site showed high divergence from CCUT1 and CCUT2 sites, compared to other CCUT‐CCUT or HEUT‐HEUT comparisons.

**FIGURE 5:**
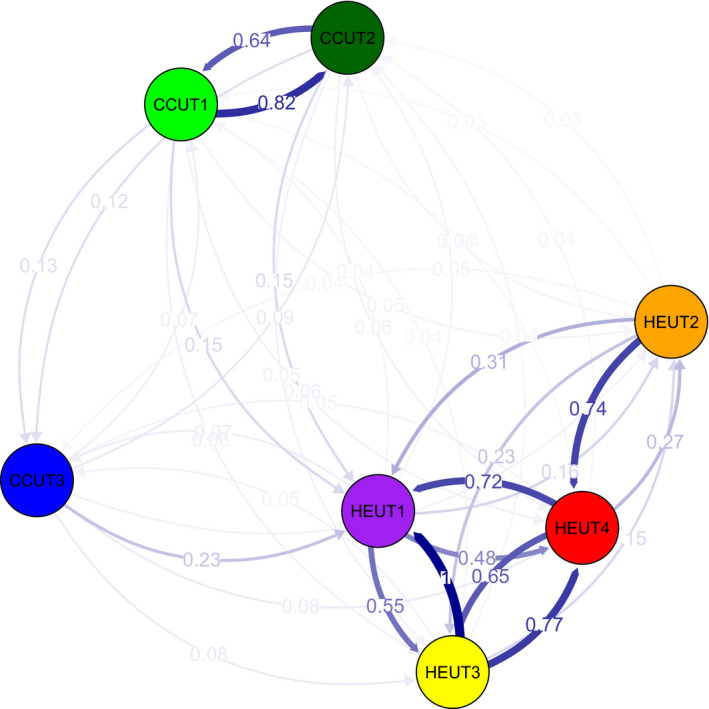
The relative rate of migration (*N*_M_) among sampling sites. Arrows indicate direction and relative rates among sites with darker blue indicating higher migration rate than lighter blue

The estimated number of migrants per generation based on the number of private alleles at each site was 0.043. The relative migration analyses conferred with our estimates of population differentiation using pairwise *F*_ST_ in that we observed high migration rates (*N*_M_) among CCUT1 and CCUT2 sites as well as among the HEUT sites (Figure 5). The CCUT3 sites showed intermediate levels of *N*_M_ among both other CCUT sites as well as the HEUT sites. Low levels of migration were observed between CCUT and HEUT sites (relative *N*_M_ ranged from 0.03 to 0.23). Overall, females and males showed comparable patterns of population differentiation (Figure 4). We observed global *F*_ST_ values of 0.43 and 0.40 for males and females, respectively.

### 3.3 Characterizing the effect of genetic drift

Using all 2955 variable SNP loci, we determined the level of genomic diversity for all seven sampling sites within CCUT and HEUT as well as each genetic cluster for *K* = 2. Overall, levels of genomic diversity (as measured by *H*_O_, *H*_E_, and *A*_R_) were higher in HEUT sites compared to CCUT sites (Table 1). Observed and expected heterozygosity was greatest in the HEUT sites, particularly in the HEUT1 site (CCUT mean *H*_O_ = 0.12, *H*_E_ = 0.10; HEUT mean *H*_O_ = 0.27, *H*_E_ = 0.25; Table 1). The highest levels of *A*_R_ were observed in the HEUT1 site (*A*_R_ = 1.86; Table 1). *F*_IS_ values ranged from −0.21 to −0.18 for CCUT sites and from −0.15 to 0.11 for HEUT sites (Table 1). All sites had significant, negative *F*_IS_ values except the HEUT4 site (*F*_IS_ = 0.11; Table 1), which had a deficit of heterozygotes that indicates individuals in these subpopulations are more related than expected. We detected private alleles in CCUT1 (*A*_P_ = 1), CCUT2 (*A*_P_ = 2), CCUT3 (*A*_P_ = 8), and HEUT1 (*A*_P_ = 17). However, when individuals were grouped into genetic clusters, a large number of private alleles were detected (Table 1). The genetic cluster that contained individuals from the HEUT sites had the largest number of private alleles (*A*_P_ = 1326). This genetic cluster also had the highest amount of genomic variation in terms of *H*_O_, *H*_E_, and *A*_R_ (Table 1), suggesting the effect of genetic drift may be more pronounced in CCUT sites.

Estimates of *N*_e_ ranged widely across sites (2.2–25.4) and had large confidence intervals (Table 1). We found that site CCUT1 had the largest effective population size at 25.4 individuals (Table 1). However, due to the presence of hierarchical structure within the HEUT4 site, it is possible estimates of *N*_e_ for this site are underestimated (linkage disequilibrium‐based methods for estimating *N*_e_ assume closed populations). We also detected significant bottlenecks (*p*‐value < 0.000001) for all sites as well as for each genetic cluster.

### 3.4 Neutral loci versus loci under selection

**FIGURE 6:**
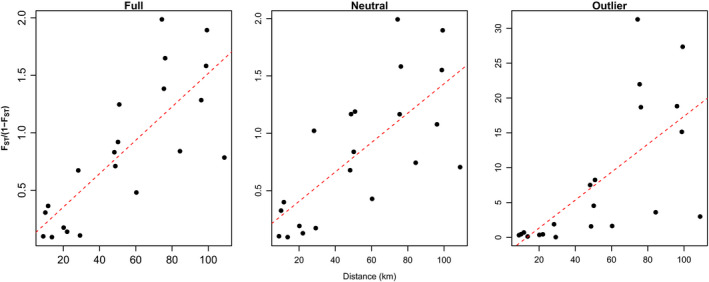
Mantel test for isolation by distance (IBD) using pairwise linearized *F*_ST_ (*F*_ST_/1 − *F*_ST_) and geographic distance (km) for all single nucleotide polymorphism (SNP) loci and site coordinates (Full; *n*_loci_ = 2955; *r* = 0.79; *p* = 0.017); only neutral SNP loci (Neutral; *n*_loci_ = 1792; = 0.72; *p* = 0.015); and only SNP loci identified as outliers (Outlier; *n*_loci_ = 51; *r* = 0.67; *p* = 0.005)

Using BayeScan, we identified 754 loci as outliers, of which, 303 loci were under divergent selection. Using PCAdapt, 531 loci were identified as under selection (Figure S9). For our outlier dataset, only loci that were identified as potentially under divergent selection in both BayeScan and PCAdapt were used (*n*_loci_ = 51; 1.7% of total loci). For the neutral dataset, all loci as identified as an outlier in either program were removed (*n*_loci_ = 1792; 60.6% of total loci). We found significant patterns of isolation by distance using the full SNP dataset (*n*_loci_ = 2955; *r* = 0.786; *p* = 0.017), only neutral loci (*n*_loci_ = 1792; *r* = 0.72; *p* = 0.015), and only outlier loci (*n*_loci_ = 51; *r* = 0.67; *p* = 0.005) based on 999 replicates (Figure 6).

The neutral loci were most similar to that of the full set, indicating that neutral loci drive the overall pattern of population divergence (Figure 3). However, the outlier loci explained the greatest total variance in genetic structure (81.7% for PCs 1 and 2) compared to either the full set of loci (50.3%) or neutral loci (46.0%), indicating that adaptive divergence also contributes to the observed patterns of genetic structure. This pattern was retained even when we subset the neutral dataset to contain an equal number of loci as the outlier dataset (by randomly sampling 51 loci; 49% for PCs 1 and 2; Figure S10). Overall, outlier loci depicted a high degree of divergence among CCUT and HEUT sites but less divergence within either the HEUT or CCUT sites. Further, CCUT3 sites clustered with other CCUT sites with outlier loci and exhibited more divergence from other CCUT sites based on neutral loci. This indicates that CCUT3 is under similar patterns of selection as other CCUT sites despite restricted gene flow.

### 3.5 Selection and environmental associations

**FIGURE 7:**
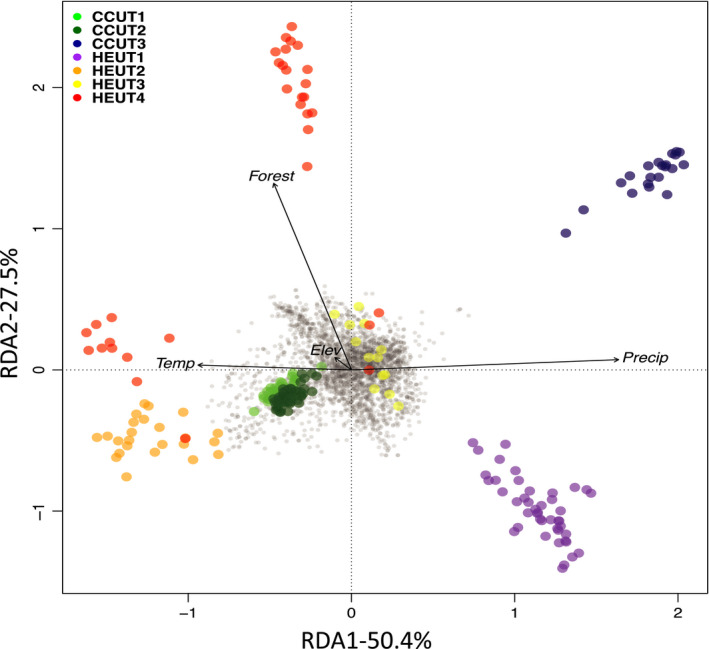
Redundancy analysis (RDA) triplots showing environmental associations with outlier loci (RDA axes 1 and 2). The dark gray dots located at the center of the plot represent single nucleotide polymorphisms (SNPs), the colored points refer to individuals (colors represent which site they were sampled from), and black vectors represent environmental variables. SNP and individual RDA scores are scaled by the square root of their eigenvalues. The direction of the arrows indicates the correlation of the environmental variable with each axis

**FIGURE 8:**
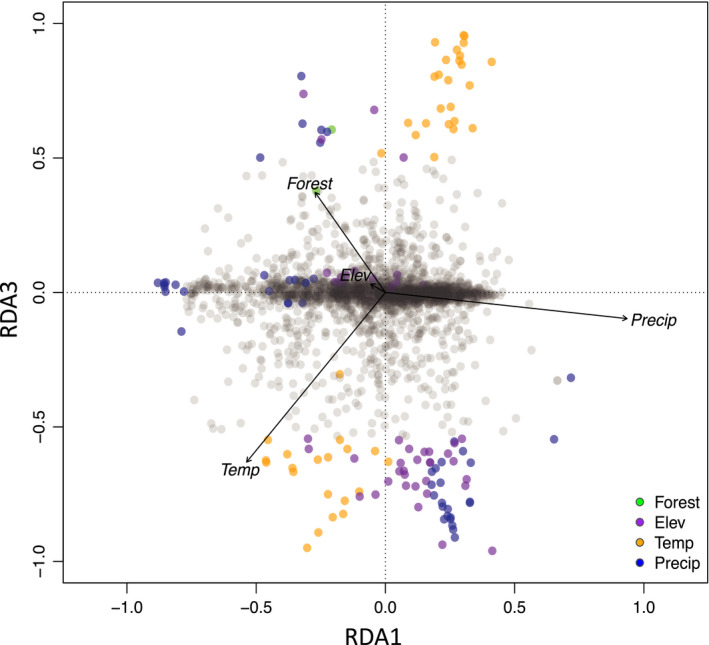
Redundancy analysis (RDA) biplots of the first and third axes show single nucleotide polymorphisms (SNPs) identified as outliers (colored circles) as well as all other SNPs (gray circles). Black vectors represent environmental variables elevation (Elev), proportion of forested land (Forest), temperature of the driest quarter (Temp), and precipitation seasonality (Precip; BIO15). The color of circles corresponds to which environmental variable had the highest correlation coefficient with each SNP. We identified a total of 141 outlier SNPs that were associated with the environmental variables (Elev = 46, Forest = 2, Temp = 45, Precip = 48). Of the outlier SNPs, 10 were identified on the first RDA axis, 107 on the third axis, and 24 on the fourth axis (axes 2 and 4 are shown in Figure S12). SNP RDA scores were scaled by the square root of their eigenvalues. The direction of the arrows indicates the correlation of the environmental variable with each axis

We found that a large proportion of the climatic variables were highly correlated (|*r*| > 0.70) to elevation. Therefore, we removed highly correlated variables as well as variables with low variation across sites. We reduced the four landscape (three landcover and elevation) and 19 climatic predictor variables to 4 total variables – proportion of forested land (Forest), elevation (Elev), mean temperature of the driest quarter (Temp; BIO9), and precipitation seasonality (Precip; BIO15). To account for the strong population structure found using STRUCTURE and DAPC, we used the individual proportion of ancestry that was assigned to the first genetic cluster when *K* = 2 (Pr(Group Membership); Figure S6) in the STRUCTURE analysis to condition the RDA – referred to as a partial RDA. Based on the ANOVA, our full model significantly explained the genetic variation in our SNP data (R^2^ = 0.09; *p* = 0.001). When we compared how well each component of the RDA explained genetic variation, we found that all four RDA axes were significant (*p* = 0.001 for all axes). The first constrained axis of the RDA explained the most variation (50.4%) followed by the second (27.5%), third (12.2%), and fourth (9.9%) axes. By using a ±3 standard deviation cutoff for SNP loadings on each RDA axis, we identified 141 outlier loci. Of the outlier loci, we found 46 SNPs were highly correlated with elevation, 2 with forests, 48 with precipitation seasonality, and 45 with temperature (Table S3). In the triplot, environmental variables (black arrows), SNPs (gray points), and individuals (colored circles) were arranged in ordination space based on their relationship with each ordination axis (Figure 7; Figure S11). From these plots, it was evident that temperature and precipitation seasonality loaded strongly on the first axis of the RDA and individuals from HEUT1 and CCUT3 showed strong associations with more precipitation and lower temperatures. Conversely, individuals from HEUT2 and HEUT4 were associated with warmer temperatures during the driest quarter and lower precipitation seasonality. Individuals from HEUT4 were strongly associated with a higher proportion of forest within a 5 km buffer while HEUT1 was more associated with a lower proportion of forest. We then looked at SNP loadings with environmental variables to see how they relate in ordination space (Figure 8; Figure S12). Of the outlier SNPs, 10 were identified on the first RDA axis, 107 on the third axis, and 24 on the fourth axis (Figure 8; Figure S12).

## 4 DISCUSSION

Through the use of high‐resolution, genome‐wide markers we have demonstrated that researchers can characterize patterns of genetic structure and gene flow, elucidate demographic processes, compare the effects of drift and selection on population divergence, and identify GEAs in a threatened species. This information is invaluable in tailoring the conservation policy of threatened species to mitigate the loss of genetic variation and maintain evolutionary potential. We found that Utah prairie dogs show high genetic structure, limited gene flow, and a lack of genetic variation across their range. This pattern can be explained sufficiently well by demographic processes– population isolation and genetic drift. These processes seem to have had a greater impact on the CCUT sites, where genetic variation was lower than in the HEUT sites. However, selection also plays a role in the divergence of Utah prairie dog populations, primarily between low‐elevation (CCUT) sites and high‐elevation (HEUT) sites.

Limited genetic variation in Utah prairie dogs puts them at risk for further erosion of variation through genetic drift. Although Utah prairie dog population sizes have either stabilized or increased within their RUs (United States Fish & Wildlife Service, 2012), these positive demographic trends contrast with our observations of limited gene flow, low effective population size, and recent genetic bottlenecks. Utah prairie dogs are highly social and have been shown to rarely leave natal colonies (Hoogland, 2013). This was reflected in both Jost's *D* and *F*_ST_ metrics. Higher rates of migration between neighboring sites combined with strong evidence of isolation‐by‐distance indicate that when prairie dogs do disperse, it is likely to nearby colonies and that long‐distance dispersal is rare or nonexistent. These combined analyses lend support to Utah prairie dogs functioning as a metapopulation with the sites acting as subpopulations within the genetic clusters. Patterns of metapopulation dynamics, largely driven by plague epizootics, have also been documented in black‐tailed prairie dog (*Cynomys ludovicianus*) colonies (Antolin et al., 2006; Sackett et al., 2012). As plague also causes high mortality in Utah prairie dogs, it may serve to drive metapopulation dynamics in these populations as well. Further, additional colonies exist between CCUT and HEUT sites. These sites could act as “stepping stones” to facilitate dispersal that would result in a stronger pattern of IBD.

The three RUs (West Desert, Paunsaugunt, and Awapa Plateau) of the Utah prairie dog are separated by physiogeographic features that could serve as potential barriers to movement, isolating populations. Our genetic structure results indicate that Awapa Plateau (where HEUT sites were located) is a distinct habitat in which individuals are isolated and diverging in allopatry, aligning well with its designation as a separate RU. In contrast, the CCUT genetic cluster encompassed sites within the Pansaugunt and West Desert RUs, indicating that these sites share historical or contemporary variation. Another study on Utah prairie dogs, that used microsatellite markers and broader geographic sampling, found Pansaugunt to be a separate genetic cluster (Brown et al., 2016). However, there was admixture between the Pansuagunt and West Desert genetic clusters. Our study and Brown et al. (2016) show agreement with low genetic variation for all sites (*H*_O_ = 0.103–0.380 for Brown et al., 2016, and *H*_O_ = 0.11–0.31 for this study) and low effective population size (*N*_e_ = 1.9–13.9 for Brown et al., 2016, and *N*_e_ = 2.1–25.4 for this study).

We did not detect evidence of sex‐biased dispersal, which we expected based on the observations in Hoogland (2013). This could be explained by a number of factors. First, young males are likely to remain in their natal coterie until they mature. If a large proportion of the males we sampled were juveniles, which was the case in another Utah prairie dog study (Brown et al., 2016), then our estimates of male dispersal could be unrealistically low. Male prairie dogs also actively defend their coterie (and females) and have even been found to occasionally engage in infanticide and cannibalism (Hoogland, 2007). This means it is possible that males disperse as observed by Hoogland (2013), but individuals that disperse experience high mortality by either other prairie dogs or by predators before they pass on their genes.

Effective population size (*N*_e_) remains an important metric for characterizing the long‐term evolutionary potential of a species, as populations with a small *N*_e_ have an elevated extinction risk due to the fixation of deleterious alleles and through the loss of adaptive variation (Jamieson & Allendorf, 2012; Phifer‐Rixey et al., 2012). Although confidence intervals for *N*_e_ values were large, it is clear that the *N*_e_ values for Utah prairie dog populations are remarkably low. Small *N*_e_ was also reported in another study of Utah prairie dog using different markers and methodology (Brown et al., 2016). Drift acts more rapidly in small versus large populations (Lacy, 1987), which means that without gene flow we should expect genetic diversity to decline quickly in Utah prairie dog populations, despite positive demographic trends. Facilitated gene flow (i.e., translocation) from populations with unique alleles and high genetic variation would increase *N*_e_ of small populations and slow drift‐based erosion of diversity (Laikre et al., 2016). However, this strategy should be pursued with caution, as maladapted individuals introduced to new environments could reduce population fitness, as shown by the low establishment success of poorly adapted seeds (Kulpa & Leger, 2013) and reduced hatching success in non‐native habitat in pike (*Esox lucius*) (Berggren et al., 2016).

Whereas restricted gene flow leaves genetic drift and inbreeding unmitigated, it also facilitates adaptation to local environments (Barrett & Schluter, 2008; Savolainen et al., 2013; Slatkin, 1987). The results from BayeScan and PCAdapt in this study reflect the observed high divergence and low rates of relative migration (*N*_M_) between high‐elevation (HEUT) sites and low‐elevation (CCUT) sites. The RDA analysis expanded on this pattern of divergence by showing that outlier loci were associated with high‐elevation environments, a greater proportion of forests, colder temperatures during the driest quarter, and greater precipitation seasonality. Differences in habitat and climate between HEUT and CCUT sites indicate that local adaptation could occur, and our outlier and GEA analyses indicate that adaptation is ongoing. Other studies have also found that the HEUT sites harbor a greater diversity of flea and small mammal species compared to CCUT sites (Bron et al., 2018; Russell et al., 2018). This variation in environment and species composition likely plays a role in plague dynamics (Eads & Hoogland, 2017; Williams et al., 2013) that could affect both population viability as well as reduce the standing genetic variation required for adaptation to occur.

A large proportion of our SNP loci in our study were identified as under divergent selection (10% using BayeScan and 18% using PCAdapt). This is somewhat larger than the proportion of SNP loci identified in studies of other mammals (7% of loci using PCAdapt in zebras (*Equus quagga*; Pedersen et al., 2018); 4.4% loci for BayeScan and 6.7% of loci for PCAdapt in red foxes (*Vulpes vulpes*; Roberts, 2019)), and likely reflects the elevated opportunity for adaptation in a species with high site fidelity, small populations, and short generation time. Genome scans can be prone to false positives and be challenging to interpret when population structure is present (Capblancq et al., 2018; Forester et al., 2016, 2018; Hoban et al., 2016). We aimed to minimize these limitations by generating a conservative set of criteria for outlier detection (those that were identified using two different approaches; 1.7% of total loci) and reducing the potential for false positives through FDR control. Further, comparing environmental variables to allele counts in a constrained ordination framework (RDA), as was done in this study, has low false‐positive rates and high true‐positive rates across demographic history and sampling schemes (Capblancq et al., 2018; Forester et al., 2016, 2018). Denser sampling across the genome, for example incorporating whole‐genome sequences, would provide a more accurate representation of genomic variation across all linkage blocks in order to better gauge local adaptation and identify loci under selection (Hoban et al., 2016; Lowry et al., 2017). Further, experimental work such as common garden or reciprocal transplant experiments would help to validate true connections between the genotypes and their associated environmental variables.

## 5 CONCLUSIONS

With continued change in land use and climate, maintaining genetic variation is of paramount importance for the survival of threatened species. Greater standing genetic variation allows for selection to act on a larger pool of phenotypic traits. Species that are most at risk of losing genetic variation and exhibiting reduced viability in the face of a changing environment are those that have: (a) small, isolated populations, (b) a history of bottlenecks/founder events, (c) low effective population size, and (d) limited gene flow among populations. Our range‐wide study on Utah prairie dogs, as well as another by Brown et al. (2016), showed that Utah prairie dogs have all the above characteristics and already exhibit limited genomic variation. We also observed signatures of selection via local adaptation that are contributing to divergence among CCUT and HEUT sites. Local adaptation reduces genome‐wide variation, but also improves a population's ability to survive in their current environment.

Translocations are currently used for prairie dogs, with considerable research focused on improving success (Curtis et al., 2014; Truett et al., 2001; United States Fish & Wildlife Service, 2012). Translocations initiate gene flow and increase the genetic variation in the recipient population (Whiteley et al., 2015), and have been successful in threatened species conservation (Bateson et al., 2014; Johnson et al., 2010). However, translocations could exacerbate population declines in threatened species by introducing maladaptive genes into populations that are locally adapted (outbreeding depression; Frankham, 2015; Weeks et al., 2011). Incorporating selection directly into translocation strategies could help to mitigate the risk of translocation‐mediated breakdown of local adaptation while increasing standing variation (Flanagan et al., 2018; Harrisson et al., 2017). We also suggest that Utah prairie dogs are low risk of outbreeding depression based on two of the three screening criteria outlined by Frankham et al. (2011) populations are chromosomally compatible, and isolation of populations occurred within the last 500 years. The third criteria for low outbreeding depression risk, that populations are not adapted to vastly different habitats, is indirectly supported by our data indicating natural gene flow between high and low‐elevation populations (HEUT sites and CCUT3) and an overall pattern of IBD. With low levels of genetic variation, especially in the CCUT sites, and limited gene flow species‐wide, the consequences of genetic drift in Utah prairie dogs may outweigh concerns of outbreeding depression from moving potentially locally adapted individuals (Hereford, 2009; Ralls et al., 2018; Weeks et al., 2016). Specifically, translocations from higher‐diversity HEUT sites to CCUT sites may serve to increase genetic variation in CCUT sites. The effect of translocations on local adaptation in CCUT is not easily predicted, but recent evidence (Fitzpatrick et al., 2020) indicates that gene flow into small, isolated populations can bolster overall genetic variation without erasing local adaptation, an ideal management outcome for Utah prairie dogs.

## CONFLICT OF INTEREST

None declared.

## Supporting information

Supplementary Material

## Data Availability

Genomic data used in this study are available from the Dryad Digital Repository: https://doi.org/10.5061/dryad.tqjq2bvxn.
