# Supplementary material for: Characterizing patterns of genomic variation in the threatened Utah prairie dog: Implications for conservation and management
Source: Evol Appl. 2020 Dec 21;14(4):1036–51. doi: 10.1111/eva.13179 (PMC8061279; doi:10.1111/eva.13179)
Supplement: Supplementary file 1 — Supplementary Material [file EVA-14-1036-s001.docx]

**Supplementary Information:**

Figure S1. Landcover (National Landcover Database; Homer, Fry, & Barnes, 2012) for each sampling location of Utah prairie dogs (*Cynomys parvidens*) in southwestern Utah, USA, was characterized using the proportion of each landcover type within a 5-km buffer area around the center of the sampling area.

Figure S2. Land cover variables (from NLCD; Homer, Fry, & Barnes 2012) and climate variables (WorldClim; Hijmans et al., 2005) for each sampling site (represented by different color) that were not used in the redundancy analysis (RDA). Definitions of WorldClim codes can be found in Table S4.

Figure S3. To reduce the presence of multicopy loci, we implemented a cut off of 2X the mode of the mean depth of coverage for each locus– (a) the number of loci at each depth prior to removing high depth loci, the red line shows the cutoff threshold for filtering (mode=17.55), (b) the mean depth of coverage for all loci prior to removing high depth loci, the red line shows the cutoff threshold for filtering), and (c) after filtering high depth loci, the final genomic dataset contained 2,955 variable single nucleotide polymorphism (SNP) loci with a mean depth of coverage of 20.08 and ranged from 7.68-35.58.

Figure S4. Mean proportion of missing loci per individual after filtering individuals with a high amount of missing data (>0.30). The minimum amount of missing data for an individual was 0.00 while the maximum was 0.26 (mean=0.04).

Figure S5. A genetic cluster (K) =2 was best supported in STRUCTURE based on (a) Ln(K) and (b) ∆K method.

Figure S6. STRUCTURE plots for (a) full run for a genetic cluster (K) =2 (most supported based on Ln(K) and the ∆K method), (b) hierarchical results for the Cedar City (CCUT) and high elevation (HEUT) genetic clusters (K=2 for each), (c) females only (K=2), (d) males only (K=2), (e) neutral loci only (K=2), and (f) outlier loci only (K=2).

Figure S7. Discriminant analysis of principal components (DAPC) showing genetic structure in Utah prairie dogs. Solutions dividing samples into 2-4 genetic clusters were informative (a) based on the Bayesian information criterion (BIC) (b).

Figure S8. Sites from the Cedar City (CCUT) area made up one genetic cluster and the high elevation site (HEUT) made up another with a genetic cluster (K) =2 in the discriminant analysis of principal components (DAPC) analysis (a). When K=4, we saw CCUT3 sites form their own cluster and some further differentiation among the HEUT sites (b). Inferred genetic clusters are indicated by the vertical lines (“inf”) and the size of boxes indicate the number individuals from each site that belong to that inferred genetic cluster.

Figure S9. Two approaches were used to identify outlier loci. A Bayesian-based program (Bayescan (Foll & Gagiotti, 2008); (a) showing the posterior odds (PO) compared to F_ST_ of each locus with a false discovery rate (FDR) threshold of 0.05) and an ordination-based method (PCAdapt (Luu, Blum, & Privé, 2019); (b) Q-Q plot showing the distribution of p-values compared to an expected uniform distribution of p-values (dark line) and (c) Manhattan plot showing the p-values for each locus). For the Bayescan results (a), loci to the right of the vertical bar on the log(PO) axis indicate loci under selection. Of those loci, the ones with positive alpha values were considered under divergent selection. Those with negative alpha values were considered under balancing selection. All loci identified as outliers in either the Bayescan or PCAdapt analysis were removed to create the neutral panel of loci.

Figure S10. Principal component analysis (PCA) to characterize genetic differentiation among Utah prairie dogs using a subset of neutral loci. (n=51). Colors correspond to sites (CCUT1, CCUT2, CCUT3, HEUT1, HEUT2, HEUT3, and HEUT4).

Figure S11. Redundancy analysis (RDA) triplots showing environmental associations with outlier loci (RDA axes 1 and 3 above and RDA axes 1 and 4 below). The dark grey dots located at the center of the plot represent SNPs, the colored points refer to individuals (colors represent which site they were sampled from), and black vectors represent environmental variables. SNP and individual RDA scores are scaled by the square root of their eigenvalues. The direction of the arrows indicates the correlation of the environmental variable with each axis.

Figure S12. Redundancy analysis (RDA) biplots of the first and third axis shows SNPs identified as outliers (colored circles) as well as all other SNPs (grey circles) (RDA axes 1 and 2 above and RDA axes 1 and 4 below). Black vectors represent environmental variables-­ elevation (Elev), proportion of forested land (Forest), temperature of the driest quarter (Temp), and precipitation seasonality (Precip; BIO15). The color of circles corresponds to which environmental variable had the highest correlation coefficient with each SNP. We identified a total of 141 outlier SNPs that were associated with the environmental variables (Elev=46, Forest=2, Temp=45, Precip=48). Of the outlier SNPs, 10 were identified on the first RDA axis, 107 on the third axis, and 24 on the fourth axis (axes 1 and 3 are shown in Fig. 8). SNP RDA scores were scaled by the square root of their eigenvalues. The direction of the arrows indicates the correlation of the environmental variable with each axis.


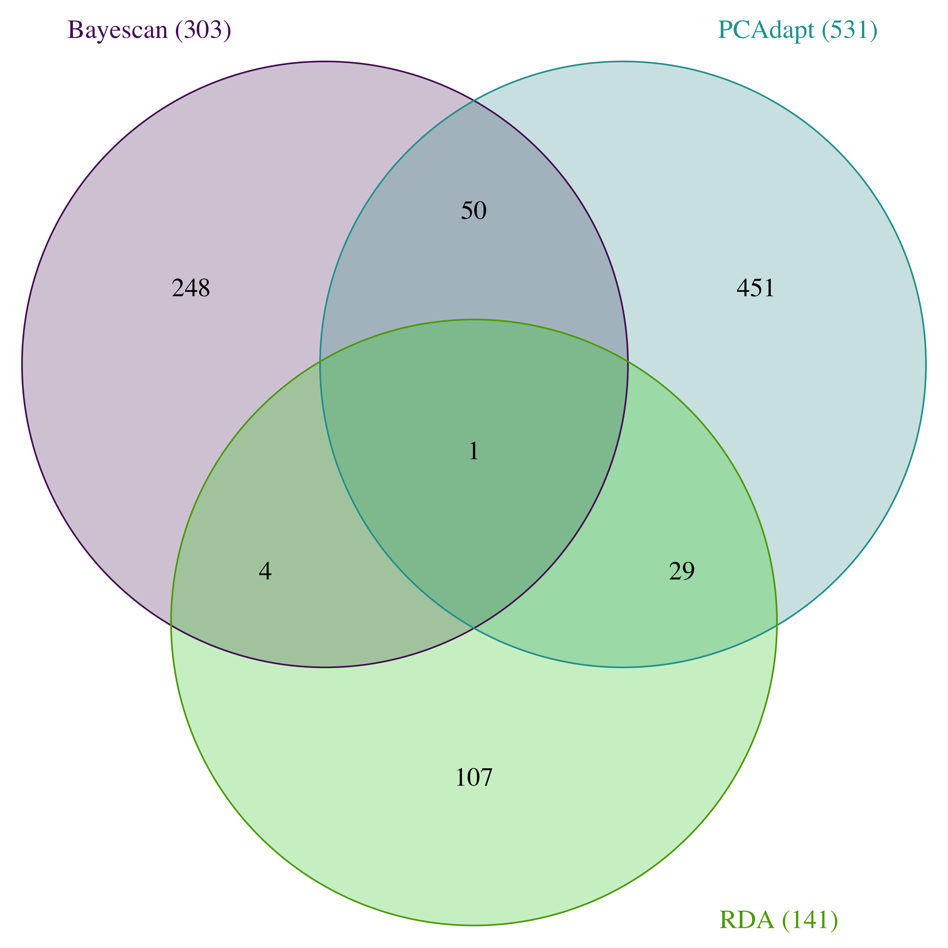


Figure S13. We used three outlier detection methods to identify patterns of selection in Utah prairie dogs (Bayescan (Foll & Gagiotti, 2008)–purple circle, PCAdapt (Luu, Blum, & Privé, 2019)-blue circle, RDA– green circle). The Venn diagram shows the number of loci that were identified for each method and the number of loci identified as outliers in multiple methods (where the circles overlap).

Table S1. Sites were between 8.91 to 108.89km apart. Sampling site size ranged from 2.95 to 14.74 ha. The distance (km) between each site is listed below the diagonal while the size of the sampling site (ha) is on the diagonal and in bold, italics.

|  | **CCUT1** | **CCUT2** | **CCUT3** | **HEUT1** | **HEUT2** | **HEUT3** | **HEUT4** |
| --- | --- | --- | --- | --- | --- | --- | --- |
| **CCUT1** | ***6.23*** |  |  |  |  |  |  |
| **CCUT2** | 29.20 | ***6.09*** |  |  |  |  |  |
| **CCUT3** | 48.70 | 28.25 | ***2.95*** |  |  |  |  |
| **HEUT1** | 108.89 | 84.37 | 60.31 | ***14.74*** |  |  |  |
| **HEUT2** | 99.26 | 74.38 | 50.87 | 10.06 | ***7.80*** |  |  |
| **HEUT3** | 98.80 | 76.19 | 50.16 | 13.69 | 11.60 | ***14.22*** |  |
| **HEUT4** | 96.12 | 75.51 | 48.17 | 22.09 | 20.16 | 8.91 | ***8.64*** |

Table S1. Sites were between 8.91 to 108.89km apart. Sampling site size ranged from 2.95 to 14.74 ha. The distance (km) between each site is listed below the diagonal while the size of the sampling site (ha) is on the diagonal and in bold, italics.

|  | **CCUT1** | **CCUT2** | **CCUT3** | **HEUT1** | **HEUT2** | **HEUT3** | **HEUT4** |
| --- | --- | --- | --- | --- | --- | --- | --- |
| **CCUT1** | ***6.23*** |  |  |  |  |  |  |
| **CCUT2** | 29.20 | ***6.09*** |  |  |  |  |  |
| **CCUT3** | 48.70 | 28.25 | ***2.95*** |  |  |  |  |
| **HEUT1** | 108.89 | 84.37 | 60.31 | ***14.74*** |  |  |  |
| **HEUT2** | 99.26 | 74.38 | 50.87 | 10.06 | ***7.80*** |  |  |
| **HEUT3** | 98.80 | 76.19 | 50.16 | 13.69 | 11.60 | ***14.22*** |  |
| **HEUT4** | 96.12 | 75.51 | 48.17 | 22.09 | 20.16 | 8.91 | ***8.64*** |

Table S3. The partial redundancy analysis (RDA) with landcover, elevation, and climatic variables identified 251 outlier loci-environment associations. We found outlier loci associated with elevation (Elev; n=46), forests (Forest; n=2), temperature of the driest quarter (Temp; n=45), and precipitation seasonality (Precip; n=48). Values below each environmental variable represent correlations. The environmental variable with the highest correlation is listed under the “Pred” column along with its corresponding correlation with each given single nucleotide polymorphism (SNP) locus.

| **Axis** | **SNP** | **Loading** | **Forest** | **Elev** | **Temp** | **Precip** | **Pred** | **R2** |
| --- | --- | --- | --- | --- | --- | --- | --- | --- |
| 1 | 56071_18.04 | -0.371 | -0.005 | 0.132 | 0.404 | -0.568 | precip | 0.568 |
| 1 | 57959_149.02 | -0.400 | 0.203 | -0.093 | 0.397 | -0.734 | precip | 0.734 |
| 1 | 84575_99.02 | 0.337 | -0.021 | -0.233 | -0.118 | 0.499 | precip | 0.499 |
| 1 | 85058_13.04 | -0.402 | 0.116 | -0.174 | 0.411 | -0.763 | precip | 0.763 |
| 1 | 133184_144.04 | -0.414 | 0.046 | 0.026 | 0.373 | -0.701 | precip | 0.701 |
| 1 | 172697_4.03 | -0.403 | 0.237 | -0.125 | 0.396 | -0.746 | precip | 0.746 |
| 1 | 179275_103.02 | -0.400 | -0.027 | 0.091 | 0.347 | -0.646 | precip | 0.646 |
| 1 | 218687_22.04 | -0.382 | 0.035 | -0.026 | 0.353 | -0.672 | precip | 0.672 |
| 1 | 256463_44.03 | -0.402 | 0.058 | 0.050 | 0.369 | -0.659 | precip | 0.659 |
| 1 | 369614_55.02 | -0.366 | 0.104 | 0.008 | 0.346 | -0.629 | precip | 0.629 |
| 3 | 47184_8.02 | 0.243 | 0.128 | 0.374 | -0.311 | -0.156 | elevation | 0.374 |
| 3 | 47881_111.02 | -0.317 | -0.258 | -0.498 | 0.403 | 0.197 | elevation | 0.498 |
| 3 | 49684_84.04 | -0.195 | -0.105 | -0.507 | 0.325 | -0.035 | elevation | 0.507 |
| 3 | 49685_68.02 | -0.196 | -0.110 | -0.503 | 0.333 | -0.048 | elevation | 0.503 |
| 3 | 49686_82.02 | -0.208 | -0.115 | -0.549 | 0.351 | -0.049 | elevation | 0.549 |
| 3 | 49687_38.04 | -0.208 | -0.115 | -0.549 | 0.351 | -0.049 | elevation | 0.549 |
| 3 | 52251_39.04 | 0.184 | 0.169 | -0.207 | -0.146 | -0.356 | precip | 0.356 |
| 3 | 52338_86.03 | -0.238 | -0.163 | 0.428 | 0.253 | 0.358 | elevation | 0.428 |
| 3 | 57961_38.03 | -0.231 | -0.163 | 0.418 | 0.225 | 0.385 | elevation | 0.418 |
| 3 | 60801_91.01 | -0.195 | -0.236 | 0.291 | 0.123 | 0.431 | precip | 0.431 |
| 3 | 64648_111.02 | 0.200 | 0.334 | 0.122 | -0.244 | -0.173 | forest | 0.334 |
| 3 | 65468_33.03 | -0.276 | -0.362 | 0.094 | 0.506 | -0.048 | temp | 0.506 |
| 3 | 67139_37.03 | -0.179 | -0.118 | 0.553 | 0.059 | 0.544 | elevation | 0.553 |
| 3 | 72382_91.02 | -0.247 | -0.208 | 0.420 | 0.248 | 0.394 | elevation | 0.420 |
| 3 | 73109_110.04 | -0.291 | -0.208 | 0.364 | 0.286 | 0.466 | precip | 0.466 |
| 3 | 73547_133.04 | 0.284 | 0.335 | 0.237 | -0.617 | 0.260 | temp | 0.617 |
| 3 | 74672_9.04 | -0.294 | -0.312 | -0.536 | 0.671 | -0.375 | temp | 0.671 |
| 3 | 74884_41.02 | -0.309 | -0.260 | 0.594 | 0.292 | 0.540 | elevation | 0.594 |
| 3 | 75811_137.01 | 0.279 | 0.310 | 0.239 | -0.615 | 0.269 | temp | 0.615 |
| 3 | 78939_146.04 | -0.300 | -0.217 | 0.379 | 0.296 | 0.481 | precip | 0.481 |
| 3 | 80478_20.02 | 0.166 | 0.169 | 0.359 | -0.409 | 0.268 | temp | 0.409 |
| 3 | 81500_61.04 | -0.237 | -0.161 | 0.392 | 0.278 | 0.317 | elevation | 0.392 |
| 3 | 82710_22.01 | -0.180 | -0.060 | -0.340 | 0.053 | 0.423 | precip | 0.423 |
| 3 | 82743_144.04 | -0.274 | -0.200 | 0.351 | 0.271 | 0.438 | precip | 0.438 |
| 3 | 83606_106.04 | 0.306 | 0.354 | 0.286 | -0.668 | 0.289 | temp | 0.668 |
| 3 | 85975_62.02 | -0.198 | -0.196 | -0.467 | 0.567 | -0.460 | temp | 0.567 |
| 3 | 88180_80.03 | 0.171 | 0.226 | 0.162 | -0.289 | 0.014 | temp | 0.289 |
| 3 | 89027_14.02 | -0.236 | -0.187 | 0.333 | 0.239 | 0.365 | precip | 0.365 |
| 3 | 89828_143.04 | 0.206 | 0.261 | 0.176 | -0.464 | 0.222 | temp | 0.464 |
| 3 | 91664_77.04 | 0.297 | 0.348 | 0.229 | -0.632 | 0.244 | temp | 0.632 |
| 3 | 92439_32.03 | 0.207 | 0.180 | -0.512 | -0.098 | -0.554 | precip | 0.554 |
| 3 | 92440_33.02 | -0.209 | -0.176 | 0.533 | 0.093 | 0.572 | precip | 0.572 |
| 3 | 93801_71.03 | -0.247 | -0.234 | -0.101 | 0.509 | -0.155 | temp | 0.509 |
| 3 | 98064_112.04 | 0.267 | 0.282 | 0.275 | -0.562 | 0.216 | temp | 0.562 |
| 3 | 106220_9.03 | -0.183 | -0.134 | 0.491 | 0.088 | 0.493 | precip | 0.493 |
| 3 | 110090_78.04 | -0.192 | -0.245 | -0.377 | 0.433 | -0.237 | temp | 0.433 |
| 3 | 119750_36.04 | -0.223 | -0.202 | 0.366 | 0.259 | 0.294 | elevation | 0.366 |
| 3 | 119751_34.02 | -0.219 | -0.199 | 0.360 | 0.255 | 0.286 | elevation | 0.360 |
| 3 | 121831_22.03 | -0.192 | -0.149 | -0.433 | 0.342 | -0.063 | elevation | 0.433 |
| 3 | 123789_131.04 | -0.276 | -0.196 | 0.342 | 0.271 | 0.444 | precip | 0.444 |
| 3 | 123865_13.03 | 0.225 | 0.230 | 0.424 | -0.526 | 0.301 | temp | 0.526 |
| 3 | 123876_15.03 | 0.207 | 0.201 | 0.430 | -0.473 | 0.263 | temp | 0.473 |
| 3 | 126307_112.01 | -0.194 | -0.205 | 0.083 | 0.310 | 0.059 | temp | 0.310 |
| 3 | 126524_19.02 | -0.215 | -0.054 | 0.274 | 0.458 | -0.105 | temp | 0.458 |
| 3 | 126530_39.02 | -0.220 | -0.099 | 0.311 | 0.456 | -0.085 | temp | 0.456 |
| 3 | 126820_89.04 | -0.208 | -0.137 | 0.219 | 0.503 | -0.209 | temp | 0.503 |
| 3 | 126831_118.03 | -0.206 | -0.120 | 0.251 | 0.496 | -0.195 | temp | 0.496 |
| 3 | 127062_142.01 | 0.260 | 0.296 | 0.247 | -0.562 | 0.236 | temp | 0.562 |
| 3 | 129015_133.04 | 0.314 | 0.361 | 0.295 | -0.684 | 0.293 | temp | 0.684 |
| 3 | 130003_90.03 | -0.185 | -0.045 | -0.283 | 0.226 | 0.143 | elevation | 0.283 |
| 3 | 130535_105.04 | -0.263 | -0.108 | 0.382 | 0.300 | 0.355 | elevation | 0.382 |
| 3 | 130547_72.04 | -0.248 | -0.117 | 0.388 | 0.347 | 0.219 | elevation | 0.388 |
| 3 | 131460_37.01 | -0.198 | -0.173 | 0.526 | 0.115 | 0.494 | elevation | 0.526 |
| 3 | 133388_122.04 | -0.258 | -0.194 | 0.339 | 0.257 | 0.409 | precip | 0.409 |
| 3 | 135744_117.02 | 0.224 | 0.168 | 0.417 | -0.412 | 0.086 | elevation | 0.417 |
| 3 | 136368_137.04 | -0.179 | -0.253 | -0.659 | 0.526 | -0.498 | elevation | 0.659 |
| 3 | 136369_121.01 | -0.192 | -0.264 | -0.655 | 0.544 | -0.490 | elevation | 0.655 |
| 3 | 136919_110.04 | -0.262 | -0.197 | 0.346 | 0.262 | 0.416 | precip | 0.416 |
| 3 | 137314_20.03 | -0.255 | -0.245 | -0.291 | 0.526 | -0.185 | temp | 0.526 |
| 3 | 139534_23.01 | -0.272 | -0.262 | -0.325 | 0.560 | -0.202 | temp | 0.560 |
| 3 | 140130_111.04 | 0.290 | 0.338 | 0.268 | -0.633 | 0.274 | temp | 0.633 |
| 3 | 144498_133.04 | 0.210 | 0.269 | 0.171 | -0.480 | 0.235 | temp | 0.480 |
| 3 | 145820_130.04 | -0.216 | -0.098 | 0.140 | 0.235 | 0.286 | precip | 0.286 |
| 3 | 154391_106.04 | -0.257 | -0.219 | 0.235 | 0.220 | 0.444 | precip | 0.444 |
| 3 | 154392_110.01 | -0.258 | -0.211 | 0.226 | 0.223 | 0.439 | precip | 0.439 |
| 3 | 156184_44.03 | 0.165 | 0.215 | -0.067 | -0.036 | -0.474 | precip | 0.474 |
| 3 | 157933_77.03 | 0.188 | 0.098 | 0.301 | -0.242 | -0.119 | elevation | 0.301 |
| 3 | 159060_11.02 | -0.219 | -0.171 | 0.462 | 0.247 | 0.321 | elevation | 0.462 |
| 3 | 161359_141.02 | -0.181 | -0.176 | -0.533 | 0.350 | -0.156 | elevation | 0.533 |
| 3 | 161473_67.04 | -0.207 | -0.198 | -0.508 | 0.289 | 0.029 | elevation | 0.508 |
| 3 | 163865_133.01 | -0.205 | -0.213 | -0.494 | 0.527 | -0.378 | temp | 0.527 |
| 3 | 167054_81.03 | -0.208 | -0.160 | 0.395 | 0.166 | 0.417 | precip | 0.417 |
| 3 | 168901_143.02 | 0.165 | 0.143 | -0.716 | -0.170 | -0.316 | elevation | 0.716 |
| 3 | 170146_142.04 | -0.244 | -0.296 | -0.266 | 0.480 | -0.128 | temp | 0.480 |
| 3 | 171629_6.01 | -0.237 | -0.199 | -0.463 | 0.312 | 0.100 | elevation | 0.463 |
| 3 | 171880_58.04 | 0.254 | 0.159 | 0.180 | -0.583 | 0.269 | temp | 0.583 |
| 3 | 179416_138.04 | -0.266 | -0.189 | 0.330 | 0.261 | 0.427 | precip | 0.427 |
| 3 | 183694_21.01 | -0.220 | -0.175 | 0.339 | 0.212 | 0.366 | precip | 0.366 |
| 3 | 184937_147.01 | 0.208 | 0.178 | 0.188 | -0.404 | 0.093 | temp | 0.404 |
| 3 | 185994_105.04 | 0.315 | 0.363 | 0.295 | -0.684 | 0.290 | temp | 0.684 |
| 3 | 193737_20.01 | 0.265 | 0.258 | 0.353 | -0.567 | 0.236 | temp | 0.567 |
| 3 | 195037_37.03 | 0.200 | 0.209 | 0.487 | -0.521 | 0.375 | temp | 0.521 |
| 3 | 195890_75.04 | 0.283 | 0.091 | 0.105 | -0.656 | 0.296 | temp | 0.656 |
| 3 | 199367_13.03 | -0.205 | -0.173 | 0.300 | 0.221 | 0.298 | elevation | 0.300 |
| 3 | 199906_109.03 | 0.307 | 0.299 | 0.392 | -0.639 | 0.247 | temp | 0.639 |
| 3 | 207526_21.03 | -0.250 | -0.222 | -0.613 | 0.549 | -0.286 | elevation | 0.613 |
| 3 | 207530_69.01 | -0.204 | -0.197 | -0.584 | 0.479 | -0.302 | elevation | 0.584 |
| 3 | 215068_104.04 | 0.228 | 0.194 | 0.041 | -0.487 | 0.149 | temp | 0.487 |
| 3 | 215134_13.02 | -0.209 | -0.175 | 0.385 | 0.246 | 0.292 | elevation | 0.385 |
| 3 | 221004_137.03 | 0.199 | 0.277 | -0.063 | -0.189 | -0.282 | precip | 0.282 |
| 3 | 221006_126.02 | 0.197 | 0.249 | -0.085 | -0.192 | -0.274 | precip | 0.274 |
| 3 | 222778_31.02 | -0.181 | -0.026 | -0.018 | 0.498 | -0.322 | temp | 0.498 |
| 3 | 223812_22.04 | -0.181 | -0.182 | -0.341 | 0.424 | -0.246 | temp | 0.424 |
| 3 | 228671_116.01 | -0.232 | 0.004 | 0.440 | 0.301 | 0.290 | elevation | 0.440 |
| 3 | 229763_113.01 | -0.278 | -0.199 | 0.347 | 0.273 | 0.446 | precip | 0.446 |
| 3 | 229962_105.02 | -0.208 | -0.041 | 0.219 | 0.292 | 0.172 | temp | 0.292 |
| 3 | 230028_125.01 | -0.233 | -0.165 | 0.288 | 0.229 | 0.375 | precip | 0.375 |
| 3 | 230647_132.04 | 0.201 | 0.054 | 0.067 | -0.486 | 0.243 | temp | 0.486 |
| 3 | 231338_64.02 | -0.249 | -0.200 | 0.352 | 0.253 | 0.387 | precip | 0.387 |
| 3 | 234279_42.04 | 0.285 | 0.278 | 0.428 | -0.632 | 0.301 | temp | 0.632 |
| 3 | 237118_95.01 | 0.265 | 0.180 | -0.507 | -0.192 | -0.574 | precip | 0.574 |
| 3 | 248099_127.01 | -0.286 | -0.204 | 0.357 | 0.281 | 0.459 | precip | 0.459 |
| 3 | 256169_108.04 | -0.229 | -0.152 | -0.445 | 0.297 | 0.112 | elevation | 0.445 |
| 3 | 267993_138.01 | -0.278 | -0.217 | 0.345 | 0.283 | 0.424 | precip | 0.424 |
| 3 | 268078_55.02 | -0.202 | -0.187 | 0.214 | 0.379 | -0.029 | temp | 0.379 |
| 3 | 314486_128.02 | 0.193 | 0.174 | 0.405 | -0.431 | 0.221 | temp | 0.431 |
| 3 | 359516_126.03 | -0.313 | -0.359 | -0.313 | 0.684 | -0.300 | temp | 0.684 |
| 4 | 47471_59.03 | 0.155 | 0.132 | -0.345 | 0.123 | -0.282 | elevation | 0.345 |
| 4 | 62490_130.04 | 0.200 | 0.210 | -0.136 | 0.223 | -0.420 | precip | 0.420 |
| 4 | 63784_14.03 | 0.172 | 0.236 | -0.273 | 0.137 | -0.264 | elevation | 0.273 |
| 4 | 76003_67.02 | 0.171 | -0.075 | -0.429 | 0.004 | -0.064 | elevation | 0.429 |
| 4 | 95643_43.03 | 0.157 | 0.162 | -0.267 | 0.076 | -0.206 | elevation | 0.267 |
| 4 | 98627_44.04 | 0.191 | 0.301 | -0.176 | 0.198 | -0.356 | precip | 0.356 |
| 4 | 107597_138.01 | 0.167 | 0.000 | -0.469 | 0.088 | -0.240 | elevation | 0.469 |
| 4 | 124844_61.03 | 0.174 | 0.344 | -0.314 | 0.178 | -0.346 | precip | 0.346 |
| 4 | 134014_97.03 | 0.165 | 0.235 | -0.313 | 0.138 | -0.310 | elevation | 0.313 |
| 4 | 144066_146.02 | -0.176 | 0.269 | -0.291 | 0.210 | -0.402 | precip | 0.402 |
| 4 | 156971_111.04 | 0.200 | 0.139 | -0.039 | 0.227 | -0.356 | precip | 0.356 |
| 4 | 166604_121.03 | 0.166 | 0.135 | -0.312 | 0.127 | -0.259 | elevation | 0.312 |
| 4 | 176605_47.03 | 0.161 | 0.192 | -0.414 | 0.125 | -0.297 | elevation | 0.414 |
| 4 | 178711_99.02 | 0.216 | 0.069 | 0.070 | 0.249 | -0.054 | temp | 0.249 |
| 4 | 186443_31.02 | 0.174 | 0.078 | -0.358 | 0.040 | -0.112 | elevation | 0.358 |
| 4 | 191751_148.02 | -0.168 | 0.340 | -0.200 | 0.223 | -0.402 | precip | 0.402 |
| 4 | 191752_33.02 | -0.168 | 0.340 | -0.200 | 0.223 | -0.402 | precip | 0.402 |
| 4 | 198663_62.02 | 0.157 | 0.204 | -0.497 | 0.050 | -0.175 | elevation | 0.497 |
| 4 | 211415_99.03 | 0.176 | 0.132 | -0.372 | 0.146 | -0.302 | elevation | 0.372 |
| 4 | 213586_44.03 | 0.179 | 0.222 | 0.158 | -0.086 | -0.157 | forest | 0.222 |
| 4 | 226233_61.04 | 0.208 | -0.222 | -0.305 | 0.041 | -0.105 | elevation | 0.305 |
| 4 | 227845_69.04 | 0.160 | 0.219 | -0.229 | 0.180 | -0.335 | precip | 0.335 |
| 4 | 227846_69.04 | 0.160 | 0.219 | -0.229 | 0.180 | -0.335 | precip | 0.335 |
| 4 | 232760_74.03 | 0.185 | -0.012 | -0.159 | 0.173 | -0.340 | precip | 0.340 |

Table S4. Definitions of the WorldClim variables that were used to detect genotype-environment associations (Hijmans et al., 2005). The final model in the redundancy analysis (RDA) included Bio09 (Mean Temperature of Driest Quarter) and Bio15 (Precipitation Seasonality (Coefficient of Variation)).

| **Code** | **Climate Variables** |
| --- | --- |
| Bio01 | Annual Mean Temperature |
| Bio02 | Mean Diurnal Range (Mean of monthly (max temp - min temp)) |
| Bio03 | Isothermality (BIO2/BIO7) (×100) |
| Bio04 | Temperature Seasonality (standard deviation ×100) |
| Bio05 | Max Temperature of Warmest Month |
| Bio06 | Min Temperature of Coldest Month |
| Bio07 | Temperature Annual Range (BIO5-BIO6) |
| Bio08 | Mean Temperature of Wettest Quarter |
| Bio09 | Mean Temperature of Driest Quarter |
| Bio10 | Mean Temperature of Warmest Quarter |
| Bio11 | Mean Temperature of Coldest Quarter |
| Bio12 | Annual Precipitation |
| Bio13 | Precipitation of Wettest Month |
| Bio14 | Precipitation of Driest Month |
| Bio15 | Precipitation Seasonality (Coefficient of Variation) |
| Bio16 | Precipitation of Wettest Quarter |
| Bio17 | Precipitation of Driest Quarter |
| Bio18 | Precipitation of Warmest Quarter |
| Bio19 | Precipitation of Coldest Quarter |
